# Using Social Media to Predict Food Deserts in the United States: Infodemiology Study of Tweets

**DOI:** 10.2196/34285

**Published:** 2022-07-05

**Authors:** Nekabari Sigalo, Beth St Jean, Vanessa Frias-Martinez

**Affiliations:** 1 College of Information Studies University of Maryland College Park, MD United States; 2 University of Maryland Institute for Advanced Computer Studies University of Maryland College Park, MD United States

**Keywords:** social media, Twitter, food deserts, food insecurity

## Abstract

**Background:**

The issue of food insecurity is becoming increasingly important to public health practitioners because of the adverse health outcomes and underlying racial disparities associated with insufficient access to healthy foods. Prior research has used data sources such as surveys, geographic information systems, and food store assessments to identify regions classified as food deserts but perhaps the individuals in these regions unknowingly provide their own accounts of food consumption and food insecurity through social media. Social media data have proved useful in answering questions related to public health; therefore, these data are a rich source for identifying food deserts in the United States.

**Objective:**

The aim of this study was to develop, from geotagged Twitter data, a predictive model for the identification of food deserts in the United States using the linguistic constructs found in food-related tweets.

**Methods:**

Twitter’s streaming application programming interface was used to collect a random 1% sample of public geolocated tweets across 25 major cities from March 2020 to December 2020. A total of 60,174 geolocated food-related tweets were collected across the 25 cities. Each geolocated tweet was mapped to its respective census tract using point-to-polygon mapping, which allowed us to develop census tract–level features derived from the linguistic constructs found in food-related tweets, such as tweet sentiment and average nutritional value of foods mentioned in the tweets. These features were then used to examine the associations between food desert status and the food ingestion language and sentiment of tweets in a census tract and to determine whether food-related tweets can be used to infer census tract–level food desert status.

**Results:**

We found associations between a census tract being classified as a food desert and an increase in the number of tweets in a census tract that mentioned unhealthy foods (*P*=.03), including foods high in cholesterol *(P*=.02*)* or low in key nutrients such as potassium (*P*=.01). We also found an association between a census tract being classified as a food desert and an increase in the proportion of tweets that mentioned healthy foods (*P*=.03) and fast-food restaurants (*P*=.01) with positive sentiment. In addition, we found that including food ingestion language derived from tweets in classification models that predict food desert status improves model performance compared with baseline models that only include socioeconomic characteristics.

**Conclusions:**

Social media data have been increasingly used to answer questions related to health and well-being. Using Twitter data, we found that food-related tweets can be used to develop models for predicting census tract food desert status with high accuracy and improve over baseline models. Food ingestion language found in tweets, such as census tract–level measures of food sentiment and healthiness, are associated with census tract–level food desert status.

## Introduction

### Background

Healthy food is vital to everyday life. However, healthy food is not equally accessible to everyone [1]. Food insecurity refers to an individual’s lack of sufficient and consistent access to healthy foods that are both affordable and good in quality because of the lack of financial and other resources [2]. In 2018, the United States Department of Agriculture (USDA) estimated that 14.3 million households (11.1%) in the United States were food insecure [2].

Geographic location is one of the most important contributing factors to food insecurity and access to healthy foods [3]. *Food deserts* can be broadly defined as geographic regions where residents do not have sufficient access to fresh fruits, vegetables, and other essential ingredients for healthy eating [4]. Access to healthy foods can be limited because of low availability of grocery stores, low access to sustainable transportation, abundance of perceivably cheaper but unhealthy fast-food options, or a combination of such reasons [5,6]. Food deserts are prevalent in rural as well as urban regions, implying that regions with an abundance of food options can still be considered food deserts based on the definition of *healthy* food [7].

### Identifying Food Deserts

The disparities in healthy food access among underserved communities have fueled the interest of public health practitioners, researchers, and community activists in not only identifying regions that are currently food deserts but also regions that are at risk for becoming food deserts in the future. The Economic Research Service at the USDA uses various indicators for the official identification of food deserts in the United States at the census tract-level. A review of the literature determined that other frequently used measures to assess food access are as follows: (1) geographic information systems (GIS) technology, where researchers use geocoding to map resources and create density maps that illustrate differences in food security and access in various locations [8]; (2) food store assessments, which may include both objective and subjective assessments of the food environment [9-13]; and (3) consumer surveys, which allow researchers to gather data from randomly selected households—data regarding household food expenditures and consumption over a specified period [4].

Although each of these food desert identification methods have been widely used and have provided rich insights into food insecurity in the United States, each method comes with unique challenges. For example, GIS technology comes with the risk of misidentification of food stores in the GIS and mapping fails to provide information about food consumption behavior [10]. Food store assessments may be associated with high costs and small, nonrandom sample sizes, as well as significant time spent conducting assessments [13]. Consumer surveys have been found to reflect self-reporting inaccuracies [14]. Each of the challenges to the state-of-the-art approaches present room for another novel approach that uses an alternative, more modern data source. This study examines the use of food ingestion language found on social media, specifically tweets, for predicting food desert status among census tracts in the United States.

### Social Media for Public Health Research

Researchers have increasingly looked to social media data as a means of measuring population health and well-being in a less intrusive and more scalable manner [15]. Social media data have proved useful in predicting health outcomes in many studies; therefore, these data may prove to be a very rich source for yet another health-related issue: food insecurity. Using social media data to predict the emergence of food deserts provides a people-centered approach for identifying food deserts by allowing for the examination of the dietary consumption and habits of individuals who reside in food deserts versus those who do not reside in food deserts [16].

Prior studies have successfully extracted information from social media to address various types of health-related outcomes, relying on the naturalistic observations deduced from social media data to answer questions related to health and well-being [17]. For example, in a study that sought to predict depression among Twitter users, researchers leveraged behavioral cues found in tweets to develop a classifier for depression [17]. In a study that considered Twitter data for various public health applications, researchers conducted syndromic surveillance of serious illnesses, measured behavioral risk factors, and mapped illnesses to various geographic regions [18]. Another study used Twitter to monitor and predict influenza prevalence in the United States by conducting a network analysis of Twitter users and demonstrating the association of social ties and colocation of people who were symptomatic with one’s risk of contracting influenza [19]. A study that sought to develop a publicly available neighborhood-level data set with indicators related to health behaviors and well-being also examined the associations between these Twitter-derived indicators and key neighborhood demographics [20]. Another study examined Instagram posts to understand dietary choices and nutritional challenges in food deserts [4]. The study by Gore et al [21] examined the relationship between the obesity rate in urban areas and the expressions of happiness, diet, and physical activity in tweets.

As seen in this study, several other studies similarly leveraged natural language processing methods such as sentiment analysis, emotion analysis, and topic modeling to use social media to answer public health research questions. For example, some studies [22-26] collected tweets over the course of the COVID-19 pandemic to examine public sentiments and opinions regarding COVID-19 vaccines. Researchers [27,28] conducted topic modeling and emotion analyses to identify the themes and emotions related to the COVID-19 vaccines to aid public health officials in the battle against COVID-19.

### Study Overview

In this study, we leveraged the linguistic constructs in food-related tweets to develop a classification model for food deserts in the United States. We considered both tweet sentiment and overall nutritional values of foods found in tweets to identify associations between living in a food desert and food consumption.

To our knowledge, this is the first study to develop a model for inferring food desert status among census tracts in the United States using Twitter data. The main objective of this study was to examine the linguistic constructs found in food-related tweets to evaluate the differences in food nutritional value and food consumption behavior of individuals in food deserts versus those in non–food deserts*.* Our key hypotheses are as follows: (1) living in a food desert is associated with positive mentions of unhealthy foods, such as tweets that mention foods that are high in caloric content or low in vital nutrients such as fiber and calcium, and (2) food ingestion language among Twitter users in a census tract can be used to infer census tract–level food desert status.

## Methods

### Overview

**Figure 1:**
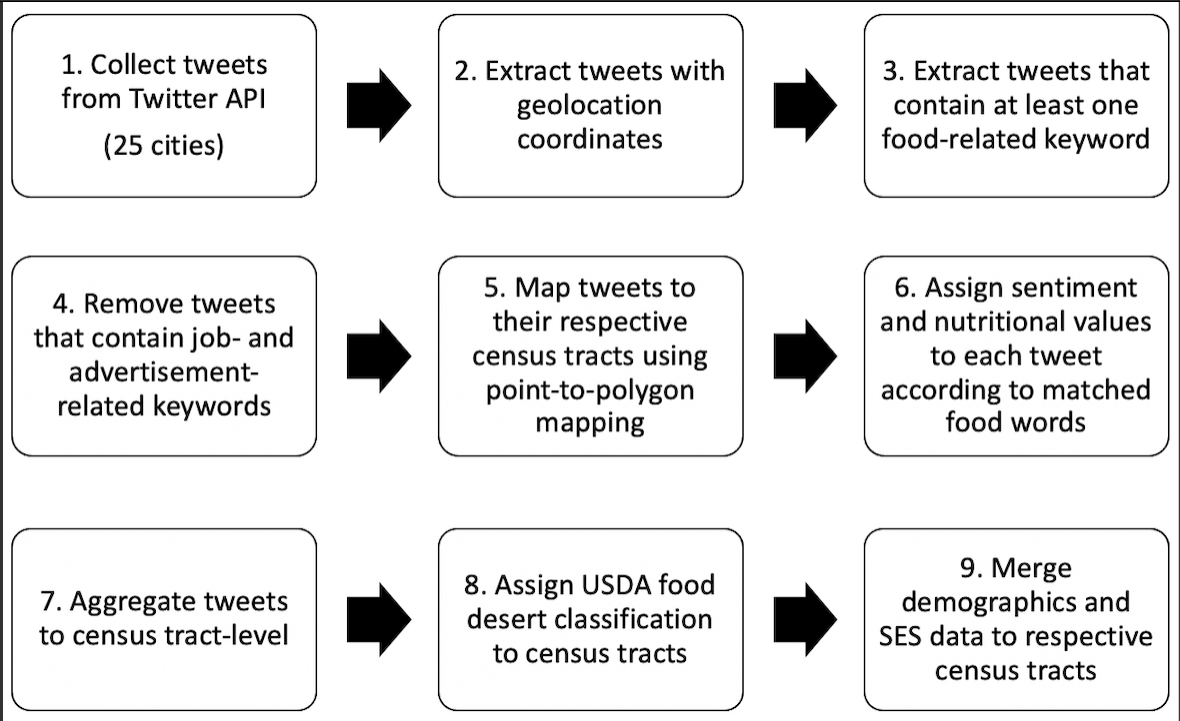
Twitter data collection process. API: application programming interface; SES: socioeconomic status; USDA: United States Department of Agriculture.

An overview of the entire data collection and preparation process is illustrated in Figure 1 and described in the following subsections.

### Data Collection

#### Twitter Data

From March 2020 to December 2020, the Twitter streaming application programming interface (API), which provides access to a random sample of 1% of publicly available tweets, was used to collect tweets (including retweets and quoted tweets) from 25 of the most populated cities in the United States (Textbox 1) [29]. The 25 cities included in this analysis are among the top 50 most populated cities in the United States. However, we decided not to go with the most populated cities (such as New York City, Los Angeles, and Houston) because we wanted to understand whether the framework we developed would be beneficial for smaller cities that are not typically the focus of these types of infodemiology studies. Public health resources directed at improving population health are historically limited and can vary from one public health jurisdiction to the next [30]. Heavily populated cities such as New York City, Los Angeles, and Houston likely have an abundance of resources (both financial and personnel) that can be used to conduct food desert identification using more traditional (expensive) methods. Although the framework outlined in this study would also be useful for heavily populated cities, we believe that less populated cities with fewer resources would benefit the most from this type of study.

When a location-based search is specified, the Twitter API extracts tweets tied to a certain location based on two criteria that are not mutually exclusive: (1) the user has their location enabled for all tweets, in which case these tweets will have specific GPS coordinates, or (2) the user has location information in their profile, such as the city and state they live in, in which case all tweets associated with this user will be tied to this location but without specific geocoordinates. In both cases, these location-tagged tweets are eligible for selection by the Twitter API when a location-based search is specified [31].

As this analysis sought to assign individual tweets to their respective census tracts, all tweets in our sample were required to have specific geolocation information (latitude and longitude GPS coordinates). A parsing module was created to filter out tweets without specific geolocation information. Next, to extract tweets related to food ingestion, tweets were further filtered by a list of 1787 food-related words from the USDA FoodData Central Database (examples are presented in Textbox 2) [32]. Names of popular fast-food restaurants extracted from Wikipedia [33] were also included in this list, as was done in the study by Vydiswaran et al [16].

Tweets related to job postings and advertisements were filtered out by excluding tweets with hashtags and keywords such as “#jobs,” “#hiring,” and “#ad.” For the purposes of this research, we assumed that the tweets in our sample, which, at minimum, contained at least one of 1787 food-related keywords, were related to food consumption, as was done in the study by Nguyen et al [20]. To assess the impact of this assumption, a random sample of 1000 tweets was selected for manual classification as food related or not food related. Among the 1000 tweets in the random sample, 770 (77%) were classified as food-related, whereas 230 (23%), although they contained food keywords, were classified as not food-related. Tweets that matched to food words but were not related to food consumption included tweets related to, for example, Apple products (eg, “I went to the Apple Store to purchase an iPhone”) and common city nicknames (eg, New York City, aka “The Big Apple”).

**Targeted cities for Twitter data collection.**

**Targeted cities for data collection**

- Albuquerque, New Mexico
- Dallas, Texas
- Atlanta, Georgia
- Baltimore, Maryland
- Colorado Springs, Colorado
- Fresno, California
- Kansas City, Missouri
- Las Vegas, Nevada
- Long Beach, California
- Louisville, Kentucky
- Mesa, Arizona
- Miami, Florida
- Milwaukee, Wisconsin
- Minneapolis, Minnesota
- New Orleans, Louisiana
- Oakland, California
- Oklahoma City, Oklahoma
- Omaha, Nebraska
- Portland, Oregon
- Raleigh, North Carolina
- Sacramento, California
- Tucson, Arizona
- Tulsa, Oklahoma
- Virginia Beach, Virginia
- Wichita, Kansas

**Examples of food-related keywords.**

**Food-related keywords**

- Healthy
- Acai
- Apple
- Apricot
- Avocado
- Banana
- Blackberries
- Blueberries
- Cantaloupe
- Cherries
- Clementine
- Unhealthy
- Cheesecake
- Cupcake
- Donut
- Pepsi
- Sprite
- Sunkist
- Red velvet cake
- Chicken McNuggets
- Double cheeseburger
- Zinger burger
- Fast-food restaurants
- Jack in the Box
- Chick-fil-A
- Burger King
- Dairy Queen
- Del Taco
- Taco Bell
- Bojangles
- Checkers
- Popeyes
- Whataburger

#### Twitter-Derived Features

We referred to similar work conducted by Nguyen et al [20] to classify each food item as healthy or unhealthy. The classification of foods as healthy or unhealthy was subjective and conducted by 2 different annotators (Daniela Nganjo and Pauline Comising). Fruits and vegetables were classified as healthy food items. Unhealthy food items included fried foods, fast-food items, and other food items commonly considered to be unhealthy. The following nutritional values, per 100 g, were obtained for each food item in the list using the USDA FoodData Central Database: calories, calcium, carbohydrates, cholesterol, energy, fat, fiber, iron, potassium, protein, fatty acids, sodium, sugar, vitamin A, and vitamin C.

To measure the healthiness of foods mentioned in tweets, the overall nutritional values of the foods mentioned in each tweet were calculated. To calculate the nutritional values of foods mentioned in each tweet, regular expression matching was used to compare the words in each tweet to the items described in the aforementioned food list (Textbox 2). The keyword-matching algorithm first searched the tweet text for matches to food keywords containing multiple words, then searched the tweet text for matches to food keywords with fewer words. Using this method, the tweet text was searched for keywords with 3 words, for example, before searching for keywords with 2 words, and the tweet text was searched for keywords with 2 words, before searching for keywords with 1 word. For example, both “Burger King” and “burger” were included in the food list. Using this keyword-matching algorithm, a tweet was searched for the keyword “Burger King” first to avoid an incorrect match to the keyword “burger” alone. Once this match was made, the keyword “Burger King” was removed from the tweet text and the remaining tweet text was searched for single-word keywords such as “burger.”

Next, the respective nutritional values for each matched food word were then calculated for the corresponding tweet. For tweets having >1 match to food names in the food list, the assigned nutritional value was equal to the average of the nutritional values for all matched food items in the tweet.

#### Sentiment Analysis

To capture the attitudes toward foods mentioned in tweets, we conducted a sentiment analysis of all tweets using the bing lexicon from the tidytext package in R [34]. The bing lexicon provides a label of *negative* or *positive* for thousands of words in the English language. To label the overall sentiment of a tweet, positive expression words were assigned a value of 1, negative expression words were assigned a value of –1, and neutral expression words were assigned a value of 0. An overall *sentiment score* was assigned to each tweet by summing the values assigned to all expression words present in a tweet. Tweets with a positive sentiment score were labeled as having overall positive sentiment, tweets with a negative sentiment score were labeled as having an overall negative sentiment, and tweets with a score of 0 were labeled as having overall neutral sentiment. The resulting tweet sentiment assignments were then used to flag the following types of tweets: tweets that mentioned healthy foods with positive sentiment; tweets that mentioned healthy foods with negative sentiment; tweets that mentioned unhealthy foods with positive sentiment; tweets that mentioned unhealthy foods with negative sentiment; tweets that mentioned fast-food restaurants with positive sentiment; and tweets that mentioned fast-food restaurants with negative sentiment. These tweet-level indicators were later aggregated to the census tract-level to produce neighborhood-specific features related to the proportion of tweets that expressed positive or negative sentiment toward healthy foods, unhealthy foods, and fast-food restaurants.

#### Mapping Tweets to Census Tracts

As this analysis examined food desert status at the census tract-level, for all census tracts in the 25 cities listed in Textbox 1, each tweet was then mapped to its respective census tract using point-to-polygon mapping of the latitude and longitude coordinates of the geolocated tweet to the bounding box of the respective census tract [35]. Once each tweet was mapped to a census tract, the tweets were aggregated to the census tract-level and the average nutritional content per food item mentioned in tweets within each census tract was calculated. Additional census tract–level food-related Twitter-derived features included the following: (1) percentage of all tweets in a census tract that mention the following with either positive or negative sentiment: healthy foods, unhealthy foods, and fast-food restaurants, and (2) average number of healthy food, unhealthy food, and fast-food mentions per tweet. Tweets with neutral sentiment were not excluded from the analysis sample, but we did not consider neutral sentiment as an independent feature. A complete list of food-related census tract–level features derived from Twitter can be found in Textbox 3.

**Twitter-derived food features.**

**Food features**

- Percentage of tweets that mention healthy foods with positive sentiment
- Percentage of tweets that mention healthy foods with negative sentiment
- Percentage of tweets that mention unhealthy foods with positive sentiment
- Percentage of tweets that mention unhealthy foods with negative sentiment
- Percentage of tweets that mention fast-food restaurants with positive sentiment
- Percentage of tweets that mention fast-food restaurants with negative sentiment
- Average number of healthy food mentions
- Average number of unhealthy food mentions
- Average number of fast-food mentions
- Average number of calories per food item (per 100 g)
- Average calcium per food item (per 100 g)
- Average carbohydrates per food item (per 100 g)
- Average cholesterol per food item (per 100 g)
- Average energy per food item (per 100 g)
- Average fiber per food item (per 100 g)
- Average iron per food item (per 100 g)
- Average potassium per food item (per 100 g)
- Average fat per food item (per 100 g)
- Average protein per food item (per 100 g)
- Average saturated fatty acids per food item (per 100 g)
- Average sodium per food item (per 100 g)
- Average sugar per food item (per 100 g)
- Average trans fatty acids per food item (per 100 g)
- Average unsaturated fatty acids per food item (per 100 g)
- Average vitamin A per food item (per 100 g)
- Average vitamin C per food item (per 100 g)
- Average number of calories per healthy food item (per 100 g)
- Average number of calories per unhealthy food item (per 100 g)

#### Food Desert Status

Once all data were collected and aggregated to the census tract-level, each census tract was classified as a food desert or not a food desert, according to the USDA Food Access Research Atlas classification of low-income and low-access tracts measured at 1 mile for urban areas and 10 miles for rural areas. The USDA classifies low-income tracts using the following criteria: (1) at least 20% of the residents live below the federal poverty level; (2) median family income is, at most, 80% of the median family income for the state in which the census tract lies; or (3) the census tract is in a metropolitan area and the median family income is, at most, 80% of the median family income for the metropolitan area in which the census tract lies [36]. Low-access census tracts are classified by a significant share (≥500 individuals or at least 33%) of individuals in the census tract being far from a supermarket or grocery store [36].

In total, 7.52% (299/3978) of census tracts with geolocated food-related tweets were classified as low-income, low-access food deserts, measured at 1 mile for urban areas and 10 miles for rural areas.

#### Demographics and Socioeconomic Status Features

Demographic and socioeconomic status (SES) characteristics at the census tract-level were pulled from the 2019 American Community Survey and merged onto the census tract–level tweets data set. The demographic variables used in this analysis are presented in Textbox 4.

**Census tract–level demographic and socioeconomic status features extracted from the 2019 American Community Survey.**

**Demographic variables**

- Percentage White and non-Hispanic
- Percentage Black or African American
- Percentage other race
- Percentage Asian
- Percentage American Indian or Alaska Native
- Percentage owner-occupied housing units
- Percentage of population living below the federal poverty line
- Number of housing units
- Number of households
- Median family income (US $, 2019)
- Median age (years)
- Population

### Data Analysis

Analyses were performed using R software (version 3.5.1; The R Foundation for Statistical Computing) and Python (version 3.8).

#### Evaluating the Association Between Living in a Food Desert and Food Ingestion Language on Twitter

To test the hypothesis that living in a food desert is associated with the food ingestion language of Twitter users, adjusted linear regression was conducted using food desert status as a treatment and the SES features listed in Textbox 4 as control features to analyze which Twitter-derived features presented in Textbox 3 were statistically significantly different between food deserts and non–food deserts. Each Twitter-derived feature (Textbox 3) was designated as the outcome variable in individual linear regression models, as specified in the following equation:

*y_Twitter_* = *β_0_* + *β_FD_x_FD_* + *β_SES1_x_SES1_* + ... + *β_SES12_x_SES12_* + *Error*

where *y_Twitter_* = Individual Twitter – derived food feature; *β_0_* = y – intercept (constant); *β_FD_*=food desert classification; and *β_SES1_*-*β_SES2_*=each of the 12 demographic and socioeconomic features listed in Textbox 4.

Twitter-derived features that were found to have individual, significant associations with food desert status were later used as features in the classification model for predicting food desert status to test the hypothesis that key food ingestion language found in tweets can be used to infer census tract–level food desert status.

#### Predicting Food Desert Status

To test the hypothesis that food ingestion language found in tweets can be used to infer census tract–level food desert status, classification models were developed using the Twitter-derived food-related nutritional features listed in Textbox 3. We developed 5 different classification models with different sets of features that would allow us to determine which models, if any, show improvements over a baseline model (Table 1). The first model, which was considered the baseline model, included demographics and SES features previously found to be strong predictors of food desert status in prior studies [37]; the second model included the demographics and SES features from the baseline model, plus the Twitter-derived food-related nutritional features presented in Textbox 3; the third model included the demographics and SES features from the baseline model, plus the tweet sentiment features; the fourth model included all the features (from models 2 and 3 combined); and the fifth model included the demographics and SES features from the baseline model, plus all Twitter-derived food-related features found to have a statistically significant association with census tract–level food desert status.

All features were standardized using minimum-maximum normalization, a method that standardizes data by rescaling the range of individual features to (0, 1), as described in the study by Cao et al [38]. The data were divided into a 70:30 training data and testing data split. Each of the models were built using 5-fold cross-validation to keep computation time to a minimum. Using the *caret* package in R, each model described in Table 1 was run using several different classification methods: adaptive boosting, gradient boosting, logistic regression, and ensemble methods [39]. The ensemble model combined adaptive boosting, gradient boosting, and logistic regression as base methods. Ensemble modeling is a process that aggregates the predictions of many different modeling algorithms and uses the results of the base models as inputs into a logistic regression model. The ensemble performs as a single model, reducing the generalization error of the prediction compared with the base models alone. The results of each classification method, regardless of performance, are presented in this paper.

**Table 1:** Classification models for predicting food desert status.

Model	Description	Features
1	Demographics and SES^a^ only (baseline)	Demographics and SES features (Textbox 4)
2	Demographics and SES+nutritional values	Demographics and SES features (Textbox 4) and Twitter-derived food-related nutritional features (Textbox 3)
3	Demographics and SES+Twitter mentions sentiment	Demographics and SES features (Textbox 4) and sentiment analysis of Twitter mentions features (Textbox 3)
4	Demographics and SES+nutritional values+Twitter mentions sentiment	Demographics and SES features (Textbox 4), Twitter-derived food-related nutritional features (Textbox 3), and sentiment analysis of Twitter mentions features (Textbox 3)
5	Demographics and SES+statistically significant features	Demographics and SES features (Textbox 4) and Twitter-derived food-related features found to have a statistically significant association with census tract–level food desert status

^a^SES: socioeconomic status.

### Ethics Approval

The University of Maryland College Park institutional review board has determined that this project does not meet the definition of human participant research under the purview of the institutional review board according to federal regulations.

## Results

### Overview

A total of 60,174 geolocated food-related tweets were collected during the data collection period. Across the 25 cities in our sample, 3978 census tracts had at least one geolocated food-related tweet, with a median of 4 (IQR 8) geolocated food-related tweets per census tract. Long Beach, California, had the largest representation of tweets (17,303/60,174, 28.75%), as well as the largest representation of users (5189/17,978, 28.86%; Table 2). Fresno, California, had the smallest representation of tweets (421/60,174, 0.7%), and Wichita, Kansas, had the smallest representation of users (132/17,978, 0.73%). The maximum number of tweets by a single individual was 1277 (from a user in Long Beach, California). On average, there were 6686 (SD 3629) tweets collected from 3264 (SD 1385) users each month. The remaining tweet and user statistics can be found in Table 2.

Table 3 displays descriptive statistics of the census tract–level Twitter-derived food features. On average, there was a higher percentage of tweets that mentioned healthy foods with positive sentiment (34%) versus negative sentiment (20%), a higher percentage of tweets that mentioned unhealthy foods with positive sentiment (34%) versus negative sentiment (17%), and a higher percentage of tweets that mentioned fast-food restaurants with positive sentiment (21%) versus negative sentiment (12%).

Table 4 displays descriptive statistics of census tract–level demographics and SES features among census tracts represented in this analysis. Across the represented census tracts, 62.7% (10,682,930/17,038,167) of all residents were White and non-Hispanic, 15.6% (2,657,954/17,038,167) were Black or African American, and 8.9% (1,516,397/17,038,167) identified as other race. The median family income across census tracts was approximately US $82,000, and the median age was approximately 37 years.

**Table 2:** Number of tweets (N=60,174) and users (N=17,978) by city.

City	Number of tweets, n (%)	Number of users, n (%)
Albuquerque, New Mexico	839 (1.39)	224 (1.26)
Atlanta, Georgia	4936 (8.2)	1739 (9.67)
Baltimore, Maryland	2521 (4.19)	684 (3.8)
Colorado Springs, Colorado	847 (1.41)	268 (1.49)
Dallas, Texas	2472 (4.11)	782 (4.35)
Fresno, California	421 (0.7)	153 (0.85)
Kansas City, Missouri	1651 (2.74)	532 (2.96)
Las Vegas, Nevada	2336 (3.88)	872 (4.85)
Long Beach, California	17,303 (28.75)	5189 (28.86)
Louisville, Kentucky	1246 (2.07)	406 (2.26)
Mesa, Arizona	1888 (3.14)	616 (3.43)
Miami, Florida	2576 (4.28)	1080 (6.01)
Milwaukee, Wisconsin	1578 (2.62)	388 (2.16)
Minneapolis, Minnesota	1282 (2.13)	471 (2.62)
New Orleans, Louisiana	2144 (3.56)	641 (3.57)
Oakland, California	2601 (4.32)	614 (3.42)
Oklahoma City, Oklahoma	1143 (1.9)	371 (2.06)
Omaha, Nebraska	742 (1.23)	198 (1.1)
Portland, Oregon	5528 (9.19)	928 (5.16)
Raleigh, North Carolina	1588 (2.64)	454 (2.53)
Sacramento, California	1721 (2.86)	565 (3.14)
Tucson, Arizona	794 (1.32)	250 (1.39)
Tulsa, Oklahoma	622 (1.03)	209 (1.16)
Virginia Beach, Virginia	960 (1.6)	212 (1.18)
Wichita, Kansas	435 (0.72)	132 (0.73)

**Table 3:** Descriptive statistics of Twitter-derived food features from geolocated food-related tweets.

Twitter-derived food features	Values, mean (SD)
Percentage of tweets that mention healthy foods, positive sentiment	33.8 (0.4)
Percentage of tweets that mention healthy foods, negative sentiment	19.8 (0.3)
Percentage of tweets that mention unhealthy foods, positive sentiment	33.5 (0.4)
Percentage of tweets that mention unhealthy foods, negative sentiment	17.1 (0.3)
Percentage of tweets that mention fast-food restaurants, positive sentiment	21.2 (0.3)
Percentage of tweets that mention fast-food restaurants, negative sentiment	11.7 (0.3)
Average number of healthy food mentions	0.2 (0.3)
Average number of unhealthy food mentions	0.4 (0.4)
Average number of fast-food mentions	0.1 (0.3)
Average number of calories per food item (per 100 g)	155.1 (96.3)
Average calcium per food item (per 100 g)	74 (91.3)
Average carbohydrates per food item (per 100 g)	23.2 (10.9)
Average cholesterol per food item (per 100 g)	57.3 (284.4)
Average energy per food item (per 100 g)	285.1 (115.7)
Average fat per food item (per 100 g)	10.4 (6.9)
Average fiber per food item (per 100 g)	1.7 (1.4)
Average iron per food item (per 100 g)	1.7 (8.5)
Average potassium per food item (per 100 g)	194.5 (93)
Average protein per food item (per 100 g)	7 (4.1)
Average saturated fatty acids per food item (per 100 g)	3.6 (2.5)
Average sodium per food item (per 100 g)	524.7 (962.7)
Average sugar per food item (per 100 g)	11.8 (8.3)
Average trans fatty acids per food item (per 100 g)	0.1 (0.2)
Average unsaturated fatty acids per food item (per 100 g)	2.6 (4)
Average vitamin A per food item (per 100 g)	548.8 (734.5)
Average vitamin C per food item (per 100 g)	7.1 (15.8)
Average number of calories per healthy food item (per 100 g)	67.4 (61.5)
Average number of calories per unhealthy food item (per 100 g)	189.8 (125.9)

**Table 4:** Descriptive statistics of census tract–level demographics and socioeconomic status features extracted from the 2019 American Community Survey.

Characteristic	Values, mean (SD)
Percentage White and non-Hispanic	62.7 (23.4)
Percentage Black or African American	15.6 (21.0)
Percentage other race	8.9 (12.3)
Percentage Asian	7.4 (9.2)
Percentage American Indian or Alaska Native	1.0 (1.9)
Percentage owner-occupied housing units	49.3 (24.8)
Percentage of population living below the federal poverty line	16.2 (12.1)
Number of housing units	1788.4 (863.5)
Number of households	1628.0 (799.1)
Median family income (US $, 2019)	82,371.4 (42,680.1)
Median age (years)	37.0 (6.8)
Population	4283.1 (2243.6)

### Hypothesis 1: Living in a Food Desert Is Associated With the Food Ingestion Language and Sentiments of Tweets Observed Among Twitter Users

The adjusted linear regression models confirmed this hypothesis, revealing significant associations between food desert status and 5 of the Twitter-derived food characteristics (Table 5). The results show that a census tract being classified as a food desert was associated with an increase in the average cholesterol concentration (per 100 g; *P*=.02*)* per food item mentioned in tweets, a decrease in the average potassium concentration (per 100 g) per food item mentioned in tweets (*P*=.01), and an increase in the average number of unhealthy foods mentioned per tweet (*P*=.03). A census tract being classified as a food desert was also associated with an increase in the proportion of tweets that mentioned healthy foods as well as the proportion of tweets that mentioned fast-food restaurants with positive sentiment (*P*=.03 and *P*=.01, respectively).

Although we did not expect to see an association between living in a food desert and an increase in mentions of healthy foods with positive sentiment, we hypothesize that such an association might reflect aspirational tweets of individuals who long for healthy food that is not present in their neighborhood (for example, the positive sentiment does not reflect food consumption but rather a wish to increase accessibility).

**Table 5:** Adjusted linear regression model results examining the associations between living in a food desert and food ingestion language of Twitter users.

Twitter-derived food features	β coefficient	*P* value	SE	R-squared
Percentage of tweets that mention healthy foods, positive sentiment	.077	.03	0.036	0.003
Percentage of tweets that mention healthy foods, negative sentiment	.023	.44	0.031	3.45×10^–5^
Percentage of tweets that mention unhealthy foods, positive sentiment	–0.051	.06	0.027	0.001
Percentage of tweets that mention unhealthy foods, negative sentiment	.022	.32	0.022	3.98×10^–4^
Percentage of tweets that mention fast-food restaurants, positive sentiment	.096	.01	0.039	0.005
Percentage of tweets that mention fast-food restaurants, negative sentiment	.010	.74	0.032	8.88×10^–5^
Average number of healthy food mentions	–0.002	.54	0.003	9.57×10^–5^
Average number of unhealthy food mentions	.014	.03	0.006	0.001
Average number of fast-food mentions	–0.003	.76	0.010	2.45×10^–5^
Average number of calories per food item (per 100 g)	.005	.58	0.009	7.93×10^–5^
Average calcium per food item (per 100 g)	–0.001	.60	0.002	7.36×10^–5^
Average carbohydrates per food item (per 100 g)	–0.009	.19	0.007	4.46×10^–4^
Average cholesterol per food item (per 100 g)	.005	.02	0.002	0.001
Average energy per food item (per 100 g)	.004	.60	0.007	7.37×10^–5^
Average fat per food item (per 100 g)	–0.005	.69	0.012	4.27×10^–5^
Average fiber per food item (per 100 g)	–0.014	.10	0.008	7.26×10^–4^
Average iron per food item (per 100 g)	–6.44×10^–4^	.56	0.001	9.04×10^–5^
Average potassium per food item (per 100 g)	–0.008	.01	0.003	0.002
Average protein per food item (per 100 g)	–0.002	.88	0.010	6.11×10^–6^
Average saturated fatty acids per food item (per 100 g)	.007	.31	0.007	2.70×10^–4^
Average sodium per food item (per 100 g)	–0.005	.06	0.002	9.13×10^–4^
Average sugar per food item (per 100 g)	–0.005	.35	0.005	2.29×10^–4^
Average trans fatty acids per food item (per 100 g)	–0.002	.79	0.007	1.78×10^–5^
Average unsaturated fatty acids per food item (per 100 g)	.002	.72	0.006	3.39×10^–5^
Average vitamin A per food item (per 100 g)	.004	.58	0.007	8.19×10^–5^
Average vitamin C per food item (per 100 g)	–5.53×10^–4^	.71	0.002	3.52×10^–5^
Average number of calories per healthy food item (per 100 g)	9.58×10^–4^	.95	0.017	1.92×10^–6^
Average number of calories per unhealthy food item (per 100 g)	.007	.64	0.015	8.42×10^–5^

### Hypothesis 2: Food Ingestion Language Among Twitter Users in a Census Tract Can Be Used to Infer Census Tract–Level Food Desert Status

To test the hypothesis that food ingestion language found in tweets can be used to infer census tract–level food desert status, we used various machine learning methods to compare the performance of 5 classification models (Table 6). In this paper, we evaluated model performance by comparing each model’s area under the receiver operating characteristic curve (AUC) metric, which measures how well each model can distinguish a non–food desert census tract from a food desert census tract. We used this metric, instead of accuracy, for evaluating model performance because this metric is better suited to measure model performance on class-imbalanced data [40], as is the case with the imbalanced food desert classification outcome in our sample data (of the 3978 census tracts, 299, 7.52%, were food desert census tracts). Model 3, which included sentiment features related to food mentions, showed an improvement over the baseline model AUC, using the gradient boosting classification method, by >7%. This was also the best performing model (AUC 0.823). Model 4, which included all Twitter-derived food-related features, showed an improvement over the baseline model AUC, using the logistic regression classification method, of nearly 19%. These results confirm hypothesis 2, suggesting that the best performing models involve the inclusion of Twitter-derived food ingestion language.

**Table 6:** Model performance.

Method and model^a^		AUC^b^
**Adaptive boosting**		
	1 (baseline)	0.759
	2	0.749
	3	0.738
	4	0.650
	5	0.723
**Gradient boosting**		
	1 (baseline)	0.766
	2	0.797
	3	0.823
	4	0.777
	5	0.699
**Logistic regression**		
	1 (baseline)	0.682
	2	0.720
	3	0.777
	4	0.809
	5	0.663
**Ensemble method**		
	1 (baseline)	0.769
	2	0.771
	3	0.760
	4	0.641
	5	0.740

^a^Model descriptions (refer to Table 1)—1: demographics and socioeconomic status only (baseline); 2: demographics and socioeconomic status+nutritional values; 3: demographics and socioeconomic status+Twitter mentions sentiment; 4: demographics and socioeconomic status+nutritional values+Twitter mentions sentiment; 5: demographics and socioeconomic status+statistically significant features.

^b^AUC: area under the receiver operating characteristic curve.

## Discussion

### Principal Findings

In this study, we sought to address two key hypotheses: (1) living in a food desert is associated with positive mentions of unhealthy foods, such as tweets that mention foods that are high in caloric content or low in vital nutrients such as fiber and calcium, and (2) food ingestion language among Twitter users in a census tract can be used to infer census tract–level food desert status. The study found significant associations between living in a food desert and tweeting about unhealthy foods, including foods high in cholesterol content or low in key nutrients such as potassium. We also found that supplementing classification models with features derived from food ingestion language found in tweets, such as positive sentiment toward mentions of healthy foods and fast-food restaurants, improves baseline models that only include demographic and SES features by up to 19%, with AUC scores >0.8.

### Study Findings in Context

Assessing and understanding the food environment in neighborhoods is key to addressing the issue of food insecurity in the United States. The USDA conducts the official identification of food deserts in the United States but this assessment is infrequent and the latest assessment from 2015 is outdated. Other methods such as GIS technology, surveys, and food store assessments, although effective, can be costly and time consuming. Although conducting assessments of food stores provides important insights into the food environment, this study suggests that perhaps residents of census tracts unknowingly provide important information regarding the food environment on Twitter through the food ingestion language found in tweets. Using social media data for food insecurity research allows researchers to examine food consumption in various regions, allowing a comparison of how food ingestion differs between areas where residents have sufficient access to healthy foods and areas where residents do not have sufficient access to healthy foods.

The findings of this study contribute to the literature on food insecurity in the United States by examining the potential effects of living in a food desert on food consumption using Twitter-derived food ingestion features as a proxy to examine food consumption. In this study, we found that food desert status is associated with not only the sentiment toward the types of foods mentioned in tweets but also the nutritional content of foods mentioned in tweets. More specifically, a census tract being classified as a food desert was associated with an increase in the average cholesterol concentration and a decrease in the average potassium concentration (per 100 g) per food item mentioned in tweets, as well as an increase in the proportion of tweets that mention unhealthy foods. A census tract classified as a food desert was also associated with an increase in the proportion of tweets that mentioned healthy foods and fast-food restaurants with positive sentiment. These findings support prior studies that also found associations between neighborhood characteristics, such as food desert status or fast-food density, and the *healthiness* of tweets in a census tract [20]. These findings also echo the findings in the study by Gore et al [21], which revealed that the prevalence of tweets containing terms related to fruit and vegetables was correlated with lower obesity rates in cities.

This study makes further contributions by examining the predictive ability of food ingestion language derived from tweets on census tract food desert status. This builds upon a similar study that used Instagram posts to understand dietary choices and nutritional challenges in food deserts [4]. In this study, we investigated to what extent ingestion language extracted from Instagram posts was able to infer a census tract’s food desert status. This study yielded a model with high accuracy (>80%).

Other similar studies that sought to examine food consumption using tweets across various geographic regions suggest that many of the food-related tweets in an area may be an artifact of visitors to the area, not residents. For example, a study conducted by Mitchell et al [41] showed that travel destinations such as Hawaii have an abundance of tweets with food-related terms. Similarly, the World Happiness Report [42] showed that a larger number of food-related words in tweets were used by users who regularly travel large distances, such as tourists. Although these studies suggest that the tweets we collected may have been from residents or from people who were simply visiting an area, in our study, we decided to consider all tweets under the premise that tweets from nonresidents can also reflect their food consumption experiences when they are in that neighborhood, which still provides some information regarding the local food environment. It is also important to note that because the data collection period for this study occurred during the height of the COVID-19 pandemic (particularly during travel restrictions and quarantine mandates), this might have allowed us to better capture local movement and tweets from actual residents in these areas because people were being encouraged to stay closer to home and not travel to other areas [43].

Developing an algorithm that predicts food deserts by extracting information from tweets allows researchers to monitor food insecurity more frequently than current methods allow. The use of tweets for research related to food insecurity provides researchers with more frequently updated information, thereby addressing the “lag between capturing information about newly opened and recently closed food retail businesses” [4]. This framework also has implications for policy making and advocacy. On the basis of the results presented in this paper, we recommend the use of similar algorithms by public health officials to encourage the allocation of food resources to census tracts that have been identified as food deserts using the algorithm, especially if these neighborhoods are not currently identified as food deserts according to the USDA’s classifications. Public health officials may also leverage this framework to advocate for policy interventions that either prevent food deserts from emerging or increase access to healthy foods in neighborhoods identified as food deserts using the algorithm, minimize the impacts of limited food access, support data-driven decision-making, and encourage grocery store chains to expand into neighborhoods based on need rather than potential profit.

### Limitations

Although prior research has proved social media to be a rich data source, it does have some limitations. The ability to pull millions of tweets from a single data source is an attractive characteristic of Twitter data, but a study conducted by Pew Research Center showed that Twitter users are more likely to be younger than the general population (29% of Twitter users are aged 18 to 29 years compared with 21% of the general population in the United States), more highly educated (42% of Twitter users are college graduates compared with 31% of the general population in the United States), have higher incomes (41% of Twitter users earn at least US $75,000 per year compared with 32% of the general population in the United States), and are more likely to consider themselves Democrats (36% of Twitter users consider themselves Democrats compared with 30% of the general population in the United States) [44]. These demographics raise some concerns in terms of bias in study results and suggest limited ability to generalize results to the larger population.

Adding to the lack of representation among Twitter users is the disparity in Twitter activity among Twitter users. The median number of tweets for Twitter users is only 2 tweets per month. Just 10% of Twitter users account for 80% of the tweets across users in the United States [44]. In studies that use Twitter data, this disparity suggests that a large sample of tweets may only reflect, in reality, a much smaller sample of individuals.

Tweets were collected using the Twitter streaming API, which is limited to a random sample of 1% of all tweets sent by Twitter users at any given time. Of this limited sample of tweets, studies have shown that only approximately 1% to 2% of the tweets from the Twitter streaming API include geolocation information [20]. Because of the nature of this study, our analysis required geolocated tweets, significantly reducing the number of tweets allowed in our sample. As a result, we excluded many census tracts in the 25 cities from our sample because of a lack of geolocated tweets that were also food related. In addition, census tracts that did contain geolocated food-related tweets may have had only a small number of tweets and these tweets may not be representative of the tweets of all Twitter users who reside in a particular census tract. As our analysis is limited to geolocated tweets, there is also the potential for tweets without location information to differ significantly from tweets with geolocation information, which may suggest biased results because of unknown underlying factors.

Despite these limitations, the results of this study confirm both our hypotheses, demonstrating that food ingestion language found in tweets provides a signal that differentiates food deserts from non–food deserts.

### Conclusions

The issue of food insecurity is an important public health issue because of the adverse health outcomes and underlying racial and economic disparities that are associated with insufficient access to healthy foods [4]. Social media data have been increasingly used to answer questions related to health and well-being. Prior research has used various data sources for identifying regions classified as food deserts [4], but this study suggests that perhaps the individuals in these regions unknowingly provide their own accounts of food consumption and food insecurity on social media. In this study, we demonstrated that food desert status is associated with food ingestion language found on Twitter and that food ingestion language can be used to predict and assess the food environment in American neighborhoods.

## Abbreviations

API: application programming interface
AUC: area under the receiver operating characteristic curve
GIS: geographic information systems
SES: socioeconomic status
USDA: United States Department of Agriculture
